# Introducing a Development Method for Active Perception Sensor Simulations Using Continuous Verification and Validation

**DOI:** 10.3390/s25247642

**Published:** 2025-12-17

**Authors:** Kristof Hofrichter, Lukas Elster, Clemens Linnhoff, Timm Ruppert, Steven Peters

**Affiliations:** 1 Institute of Automotive Engineering, Technical University of Darmstadt, Otto-Berndt-Straße 2, 64287 Darmstadt, Germany; steven.peters@tu-darmstadt.de; 2 Persival GmbH, Carlo-Mierendorff-Strasse 2, 64372 Ober-Ramstadt, Germany; lukas.elster@persival.de (L.E.); clemens.linnhoff@persival.de (C.L.); timm.ruppert@persival.de (T.R.)

**Keywords:** credible perception sensor simulation, simulation development, sensor model validation, virtual validation, lidar simulation

## Abstract

Simulation-based testing is playing an increasingly important role in the development and validation of automated driving functions, as real-world testing is often limited by cost, safety, and scalability. An essential part of this is the simulation of active perception sensors such as lidar and radar which enable accurate perception of the vehicle’s environment. In this context, the particular challenge lies in ensuring the credibility of these sensor simulations. This paper presents a novel method for the efficient and credible realization and validation of active perception sensor simulations in the context of the overall development process. Since the validity of these simulations is crucial for the safety augmentation of automated driving functions, the proposed method integrates a continuous verification and validation approach into the development process. Using this method, requirements like individual sensor effects are iteratively implemented into the simulation. Every iteration ends with the verification and validation of the resulting simulation. In addition, initial practical approaches are presented for validating measurement data required for the development process to avoid errors in data acquisition and for deriving quantified acceptance criteria as part of the validation process. All new approaches and methods are subsequently demonstrated on the example of a ray tracing-based lidar sensor simulation.

## 1. Introduction

Automated driving (AD) offers transformative potential across different domains of transportation. However, validating AD functions solely through real-world testing is impractical due to cost, scalability, and safety concerns. As a result, simulation-based approaches have emerged as a fundamental tool for systematic and reproducible testing [1] (pp. 443–446). A crucial component of any automated vehicle is the perception system, which relies, among other sensors, on active perception sensors such as lidar and radar to build a detailed representation of the environment, forming the basis for higher-level understanding and decision-making [2]. Therefore, simulation-based testing like software-in-the-loop requires sufficiently realistic perception sensor simulations in order to replicate the vehicle’s environment perception. From a regulatory perspective, simulation-based approaches are also becoming increasingly important in the context of validating AD and Advanced Driver Assistance Systems (ADASs). UN Regulation No. 171 from the United Nations Economic Commission for Europe (UNECE) considers virtual testing to be part of the validation of ADAS [3] (p. 27). Similarly, other UNECE guidelines regard virtual testing as an essential component in the validation of AD systems and their safety, complementing real-world and track testing ([4] p. 2, [5] p. 5). The Commission Implementing Regulation (EU) 2022/1426 of the European Commission also proposes simulations to verify the safety concept of AD systems [6] (p. 34). All of the aforementioned regulations and guidelines require assessing the credibility of the virtual tool chain to ensure the quality and reliability of the simulation results ([3] p. 42, [6] p. 34, [4] p. 10, [5] p. 13). The Regulation (EU) 2022/1426 states, for example, that *“manufacturers shall demonstrate the scope of the simulation tool, its validity for the scenario concerned as well as the validation performed for the simulation tool chain (correlation of the outcome with physical tests)"* [6] (p. 34). In this context, a variety of additional requirements for the manufacturer are defined as part of the credibility assessment [6] (pp. 50–59). For example, the manufacturer must document the data used to calibrate and validate the models and document quality characteristics [6] (p. 54). The tolerance for the correlation between simulation and reality must also be justified within the scope of validation, and known limitations and uncertainties and their sources must be recorded [6] (p. 55). Together with the other requirements specified in the regulations and guidelines, this highlights the necessity for sophisticated methods in the development and validation of perception sensor simulations. Such methods are essential to enable credible simulations for the virtual validation of safety-relevant ADAS and AD functions.

### 1.1. State of the Art on Active Perception Sensor Simulation and Validation Methodology

Schlager et al. [7] provide a comprehensive state-of-the-art overview and collection of existing active perception sensor models in the context of automated driving. In their work, sensor simulation models are categorized based on their level of fidelity into low-, medium-, and high-fidelity models. Low-fidelity models rely on abstract object lists and mainly represent geometric aspects such as field of view and occlusion. Medium-fidelity models extend this by incorporating physical effects and detection probabilities. In sensor models of high fidelity, raw data output is computed through rendering techniques such as ray tracing based on 3D environment data supplied by the environment simulation. While high-fidelity simulation models provide the most accurate and detailed reproduction of real sensor behavior, they also require the greatest computational effort. Following this classification, Schlager et al. provide a structured overview of existing sensor simulation models for lidar, radar, and camera, highlighting their underlying principles, strengths and limitations [7] (pp. 238–255). Rosenberger expands this collection in his dissertation by including additional radar and lidar simulation models [8] (p. 26). An alternative categorization of sensor simulation models is proposed by Rosenberger et al. [9], who differentiate models based on their underlying modeling principles. These include ground truth models, idealized, phenomenological, stochastic, and physical models, each offering varying degrees of accuracy and complexity. Ground truth models simply convert world coordinates into sensor coordinates without modifying the information. Idealized models go a step further by simulating a noise-free sensor that neglects physical effects like occlusion or signal degradation. In contrast, phenomenological models combine stochastic and physical modeling to reflect observable sensor effects more realistically. Purely stochastic and physical models rely either on data-driven approaches or physical signal descriptions, respectively.

In addition to their own lidar sensor model, Haider et al. [10] (pp. 4–6) also provide a collection of state-of-the-art lidar models, including open-source and commercial tools such as CARLA [11], CarMaker from IPG Automotive GmbH [12], DYNA4 from Vector Informatik GmbH [13], AURELION from dSPACE GmbH [14] and Virtual Test Drive from Hexagon AB [15]. Further commercial tools for simulating perception sensors are PROSIVIC from ESI Group [16,17], Ansys AVxcelerate Sensors from Ansys, Inc. [18], SCANeR from AVSimulation [19], AVL Scenario Simulator from AVL List GmbH [20] and Simcenter Prescan from Siemens Digital Industries Software [21]. The dissertations of Schmidt-Kopilaš [22] (p. 38) and Ngo [23] (pp. 23–29) as well as the survey by Magosi et al. [24] each contain a further collection of existing sensor models, with the latter two focusing mainly on radar models. Among the lidar sensor models commonly cited in several of the mentioned collections are, for example, those proposed by Hanke et al. [25], Li et al. [26], Zhao et al. [27], Goodin et al. [28], Gschwandtner et al. [29], Fang et al. [30], and Bechtold and Höfle [31]. Commonly cited radar models are those of Hirsenkorn et al. [32], Holder et al. [33], Bernsteiner et al. [34], Cao [35], Schuler et al. [36], and Wheeler et al. [37]. A number of further models can be found in the mentioned collections.

In general, most publications cover one or more aspects within the overall context of developing and validating perception sensor models. The works of Hanke et al. [38], Rosenberger et al. [39,40], Linnhoff et al. [41] and Schmidt et al. [42], for example, focus on the decomposition and modularization of perception sensors. They further address the derivation of structured simulation architectures and the definition of corresponding interfaces. In this context, the ASAM Open Simulation Interface (OSI) [43,44] and the Functional Mock-up Interface (FMI) standard [45] are often used for communication between the individual architectural components. Other useful formats and standards for simulating perception sensors or AD systems/ADAS in general are, for example, ASAM OpenMATERIAL 3D [46], OpenSCENARIO XML [47] and OpenDRIVE [48], as well as Lanelet [49,50], CommonRoad [51], Scenic [52] and SUMO [53].

Another important aspect in the context of developing sensor models is the definition of requirements and the specification of the model. These requirements are essential because they define the evaluation criteria for validation and also set the limits of the scope of validity. To support the derivation of requirements, Holder et al. [54] propose a data-driven approach to identify features in lidar point clouds that are relevant for object classification. In his dissertation, Hirsenkorn [55] defines a number of rather high-level requirements regarding, for example, the completeness, scalability, traceability, and interfaces of a sensor simulation. Rosenberger et al. [9] (pp. 5–6) present a five-step approach for defining requirements for sensor simulations. The details of these steps are described in Section 2.2.1 below. A basis for the specification of sensor models in the form of a collaborative collection of cause–effect chains is introduced by Linnhoff et al. [56]. In addition, a method for evaluating the relevance of individual cause–effect chains is also presented. Incorporating the work of Linnhoff et al., Schmidt-Kopilaš [22] (pp. 69–109) develops a comprehensive specification method for sensor models in his dissertation. With this method, for example, the fidelity of the simulation, the sensor technology and the inputs and outputs are defined as part of a rough specification. A detailed specification then covers the selection of sensor effects and their integration into the simulation architecture.

In order to use sensor simulations for the development and validation of AD and ADAS functions, it is essential to ensure that they are valid for the intended usage ([9] p. 1, [7] p. 257). Accordingly, many publications are concerned with the verification and validation (V&V) of sensor simulations. In their dissertations, Rosenberger [8] (pp. 42–73) and Elster [57] (pp. 17–21) each describe and analyze several relevant methodologies specifically for V&V of active perception sensor simulations. One of these methodologies is that of Schaermann [58], which builds on methodologies proposed by Sargent [59], Oberkampf and Trucano [60] and Roth et al. [61]. Furthermore, the methodologies introduced by Ngo [23,62], Holder [63] (pp. 39–40), Eder [64] and Magosi et al. [65] are mentioned, as well as a more generic approach by Riedmaier et al. [66]. They all include, among other things, comparisons of real and simulated data, whereby the differences are quantified using different metrics. To some extent, the aspect of uncertainties during the validation process is also taken into account. Beyond that, further studies, such as those conducted within the PRISSMA project, present similar approaches for the V&V of radar and lidar simulations [67] (pp. 28–71). Fonseca i Casas [68] presents a further high-level, generically applicable methodology that focuses specifically on dealing with assumptions. Another comprehensive and detailed methodology, initially developed by Viehof [69] for validating vehicle dynamics simulations and later adapted by Rosenberger et al. [9] for perception sensor simulation, addresses numerous different aspects, including requirement definition and uncertainty considerations. Consisting of six different stages, the methodology begins with the definition of requirements, which is followed by the design of the validation study. In the third stage, the preparation of data acquisition takes place, followed by the actual acquisition and analysis of that data in the fourth stage. This includes both the collection of real-world validation data and the generation of corresponding simulated data. The fifth stage comprises an iterative validation process, in which not only the simulation model itself, but also the validation data and scenarios are examined. Finally, stage six concludes with an evaluation of the overall validity. A broader perspective on simulation-based product development is presented by Ahmann et al. [70], who propose a domain-independent approach for the continuous assessment of simulation credibility. In context of the Credible Simulation Process framework proposed by Heinkel and Steinkirchner [71], which embeds credible simulation within the overall product development process, Ahmann et al. introduce so-called credibility levels as one element to assess simulation trustworthiness. In the course of presenting new lidar or radar models, modeling principles or other related aspects, certain works also provide exemplary validation approaches (e.g., [10,25,27,30,72,73]). However, while Schlager et al. highlight in their state-of-the-art analysis that several sensor models have been validated against real-world data in some way, others lack such validation [7] (pp. 244–257).

### 1.2. Key Contributions and Structure

In this context, it should be made clear that, according to the understanding of the authors, comparing real and simulated data does not necessarily imply that the sensor simulation is suitable for validating safety-relevant ADAS and AD functions. Instead, the entire development process must be considered, and factors such as the definition of requirements and acceptance criteria must also be taken into account, especially considering the regulations and guidelines of the UNECE and European Commission that impose extensive requirements regarding the credibility of simulations. For this reason, this paper takes a holistic view of the development and validation process for sensor simulations, starting with the definition of requirements and ending with the validation result. In particular, the connections between the requirements, the test cases with acceptance criteria, the development and implementation of the simulation model, and the final validation will be addressed, which is rarely the case in the existing literature. Furthermore, the development process is not considered from a high-level perspective, but rather from the practical point of view of a developer or supplier. The reason is that, although the methodology proposed by Viehof [69] and Rosenberger et al. [9] offers a valuable overarching framework regarding the validation of sensor simulations, its practical application reveals certain shortcomings. For example, it is unclear how exactly real measurement data can be validated in practice. Furthermore, a question arises regarding how to systematically derive test cases for (V&V) from the specified sensor simulation requirements to establish a direct connection between the two. Another aspect that has yet to be addressed is how quantitative acceptance criteria for the derived test cases can be argued. This paper presents and discusses approaches to address these issues, as these are the prerequisites for the key element of this work. The key innovation is an iterative, effect-, cause-, and function-based development method for sensor simulations that incorporates continuous V&V. The focus is not just on maximizing transparency and traceability in order to come closer to credibility in terms of the regulations and guidelines, but also on efficiency with automation in mind. This new method provides a practical and efficient implementation of Viehof’s and Rosenberger et al.’s methodology in the broader context of simulation development, distinguished, in particular, by the continuous V&V approach. Furthermore, the method ensures direct integration of V&V into the sensor simulation development process. To show that the new approaches and methods are applicable in practice, they are exemplarily demonstrated by developing a simple ray tracing-based lidar sensor simulation. In summary, the process presented in this paper is intended to be a first step towards a complete development process for credible lidar and radar sensor simulations in line with the regulations and guidelines of the UNECE and European Commission. Moreover, the process explicitly reflects the perspective of the simulation developer or supplier, while end-user V&V represents a different use case and lies outside the scope of this work. The following key contributions are made:

- An iterative, effect-, cause-, and function-based method for efficient development of traceable sensor simulations with continuous V&V.
- Method for deriving test cases from the simulation requirements and a taxonomy for structuring validation test cases.
- Approaches towards the validation of data acquired for the development and V&V process.
- Demonstration of an approach for the systematic, empirical derivation of acceptance criteria for validation test cases.

This publication is divided into three main parts. First, the novel method, including the necessary preparatory steps, is presented in Section 2. Subsequently, the method is applied in Section 3 to develop and validate an exemplary lidar sensor simulation. Finally, the discussion in Section 4 addresses limitations and further open research questions.

### 1.3. Definition of Terms

To avoid misunderstandings, important terms that are used in due course are defined below. According to Oberkampf and Trucano [60] (p. 719), the term verification describes the *“process of determining that a model implementation accurately represents the developer’s conceptual description of the model and the solution to the model"*. Validation, in contrast, refers to *“the process of determining the degree to which a model is an accurate representation of the real world from the perspective of the intended uses of the model"* [60] (p. 719).

An effect is defined, according to Linnhoff et al. [56] (p. 2), as the *“deviation from the originally existing information about the environment in the signal or data."* A condition leading to this deviation is referred to as the cause of an effect [56] (p. 2).

In the context of lidar sensors, a detection is defined in accordance with DIN SAE SPEC 91471:2023-05 [74] (p. 8) as a measured signal of the sensor. An alternative, more general definition can be found in the ISO 23150:2023 [75] (p. 2). A signal is a *“single distance information at a certain azimuth and elevation which may as well contain intensity, width, noise floor and other parameters"* [74] (p. 8). A set of detections in a 3D coordinate space is referred to as a lidar point cloud [74] (p. 7). Further based on DIN SAE SPEC 91471:2023-05 [74] (p. 9), the term frame is defined as a periodical scan of the full sensor field of view.

In the context of this work, the term measurement refers to the process of capturing data. A recording is chronologically connected data originating from a single specific measurement, such as a consecutive set of frames from a lidar sensor. A further distinction is made between real, simulated and reference measurements, which generate real, simulated and reference data, respectively. Real measurements refer specifically to data acquired using the real perception sensor. Measurements conducted with the virtual perception sensor within the simulation are referred to as simulated measurements. Any measurements performed from sensors other than the perception sensor are called reference measurements [8] (p. 7). This includes, for example, the measurement of object positions in the real-world measurement setup. As real data from the perception sensor is used for both calibration and validation of the sensor simulation, it is further necessary to distinguish between calibration and validation measurements.

## 2. Proposed Continuous V&V Development Method for Sensor Simulations

The overall structure of the subsequent simulation development and V&V process is derived from and aligned with the methodology proposed by Viehof [69] and Rosenberger et al. [9]. The section begins with a description of the basic simulation architecture, which has been presented in previous works by the authors ([76] p. 3, [77] p. 22). Next, the necessary preparatory steps are explained. They consist largely of existing research. Novel in this context is a method for deriving test cases (Section 2.2.2) and a taxonomy for structuring those (Section 2.2.3). Furthermore, the aspects relating to data acquisition in Section 2.2.4 are also new. The key novelty, an iterative development method, is presented in Section 2.3. Some of the ideas and approaches presented were originally introduced in the main author’s master’s thesis [77] and are further summarized, refined, and extended.

### 2.1. Sensor Simulation Architecture

**Figure 1:**
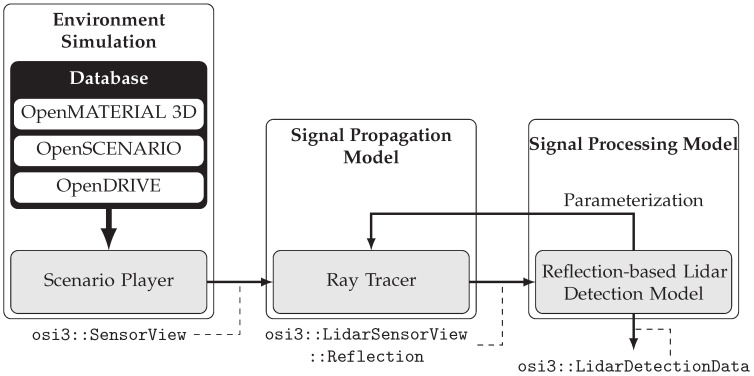
Basic architecture of the exemplarily developed lidar sensor simulation. Based on [76] (p. 3, © SAE International) and [77] (p. 22).

The simulation architecture is necessary to introduce and clarify the proposed method and enables its later application for the exemplary development of a lidar sensor simulation. Figure 1 shows the architecture, which has been presented in a similar form in previous works by the authors ([76] p. 3, [77] p. 22). Fundamentally, it consists of the three components environment simulation (ES), signal propagation model (SPG), and signal processing model (SPC). The architecture’s modular design supports flexible development and simplifies V&V by allowing components to be handled separately. To ensure that the individual modules are compatible with each other, standardized interfaces are essential. For this purpose, all components of the simulation utilize OSI [43,44]. Each component can be packaged as a Functional Mock-up Unit as part of a co-simulation, exchanging OSI data via the FMI standard [45].

In the environment simulation, environment data, provided for example in the form of the ASAM OpenMATERIAL 3D [46], OpenSCENARIO XML [47] and OpenDRIVE [48] standards, is processed to generate ground truth information (osi3::SensorView). Subsequently, this information is passed on to the signal propagation model. In the signal propagation model, a 3D environment is generated in every frame based on the provided ground truth data. The emitted laser light is simulated as it propagates through and interacts with the surrounding environment. Therefore, general physical aspects of light propagation that are not sensor-specific are covered within this model. This model utilizes a ray tracing approach, implemented in the Sensor Model Development Library (SMDL) by Persival GmbH [78]. The signal processing model takes the reflection data (osi3::LidarSensorView::Reflection) generated by the ray tracer and simulates the internal processing steps of the sensor, resulting in a point cloud output in form of osi3::LidarDetectionData. Since parameterizing the ray tracer is seen as part of the signal processing model, all sensor-specific characteristics, including the wavelength or beam divergence of the laser light, are specified in this model. Furthermore, all available interfaces for accessing data from the real sensor are also modeled in the signal processing model. According to the definition of Rosenberger et al. [9] (p. 3), the architecture presented is suitable for physical-based simulations due to the ray tracing approach. Following Schlager et al. [7] (p. 239), the term high fidelity also applies. The signal processing model is specifically configured for lidar sensors, as this sensor type is used in the example developed in this work. Nevertheless, the modular architecture allows for straightforward adaptation to radar simulations as well. The new development method introduced later is not restricted to the architecture presented in Figure 1 and can be applied to other simulation architectures as well.

### 2.2. Preparatory Steps for Simulation Development and V&V

The preparatory steps are essential, as they lay the foundation for applying the new method by gathering all necessary input information and data required for the development and V&V process. First, the requirements for the sensor simulation are defined. These determine what needs to be developed and specify, among other things, the intended functionality of the simulation. To make the requirements assessable in the context of V&V, corresponding test cases with acceptance criteria are derived next. The final preparatory step addresses the acquisition of data needed for the development and V&V of the sensor simulation. These preparatory steps are based on existing approaches, which are partly supplemented and refined by the authors. The underlying structure is again derived from the methodology proposed by Viehof [69] (p. 47) and Rosenberger et al. [9] (pp. 10–11), as it includes most of the steps described.

#### 2.2.1. Definition of Requirements

The requirements for the sensor simulation are needed, on the one hand, to determine what exactly needs to be developed and, on the other hand, provide criteria to assess the final simulation. Consequently, this allows a statement to be made about the success of the development process. The following section is based on a step-by-step approach proposed by Rosenberger et al. [9] (pp. 5–6) as well as approaches from the work of Linnhoff et al. [56] (pp. 4–5) and Schmidt-Kopilaš [22] (pp. 69–109).

Fundamentally, it is distinguished between internal and external requirements [77] (pp. 26–28). A similar distinction is proposed by Schmidt-Kopilaš [22] (p. 73). In the context of this work, external requirements refer to specifications defined by external stakeholders, such as the customer or other fixed constraints [77] (p. 26). This includes software and hardware requirements as well as requirements regarding the use of certain standards and availability of real sensor interfaces. Also, part of the external requirements are the inputs and outputs of the simulation mentioned by Schmidt-Kopilaš [22] (p. 73), as well as the desired fidelity.

Internal requirements, as defined in this work, specify which characteristics of the real sensor or the environment must be represented by the virtual sensor [77] (p. 26). To determine these internal requirements the approach of Rosenberger et al. [9] (pp. 5–6) is used. Accordingly, the output interface of the simulation is examined first, followed by selecting relevant sensor effects from an existing collection. As a collection of sensor effects—for example, the Perception Sensor Collaborative Effect and Cause Tree (PerCollECT) introduced by Linnhoff et al. [56] (pp. 4–5)—can be utilized. PerCollECT is a tree-based collection of sensor effects and causes mainly designed for lidar, radar, and camera. Within the tree structure, a distinction is made between the three categories of sensor effects, design parameter causes that are sensor-specific and sensor-independent causes. This classification is also applied in the context of this paper. PerCollECT does not provide a quantitative description of effects and causes or a specific definition of interfaces. Both must be added after selecting the individual effects and causes in order to apply the subsequent method. Quantitative descriptions allow the evaluation of effect- and cause-related requirements via metrics, while interface definitions ensure that the effects and causes are detectable at the relevant sensor outputs. The selection of possible effects and causes depends on and is partly limited by external requirements ([22] pp. 73–74, [77] p. 26). An example for this is the available interfaces of the real sensor, as specified in the external requirements. To be able to validate certain sensor effects and causes, the interfaces at which they are observed at the real sensor must be accessible. An effect or cause that is not observable in the sensor’s output data cannot be validated [77] (p. 26). To obtain this specific selection, according to Rosenberger et al. [9] (pp. 5–6), the relevance of the effects and causes shall be assessed in consideration of a specific downstream system under test (SuT). For this purpose, the Cause, Effect and Phenomenon Relevance Analysis (CEPRA) method introduced by Linnhoff et al. [56] (p. 5) can be used, as it provides a structured tool for evaluating the relevance of effects and causes with respect to a specific sensor and SuT. This SuT could be, for example, a specific automated driving function. For the application, two experts are needed to assess the occurrence of the effects and causes in a defined operational design domain (ODD) and the impact on the SuT. An ODD defines the operating conditions under which the real sensor is designed to function [79] (p. 15). An alternative approach is a sensitivity analysis along the lines of Holder et al. [54] and Ngo et al. [80], in which the influence of individual effects and causes on a particular SuT is examined. Effects and causes that have the greatest influence on the SuT are therefore prioritized for implementation.

For the new development method, a third category called sensor functions is added alongside effects and causes. According to ISO/IEC/IEEE 24765:2017 [81] (p. 190), a function is a *“defined objective or characteristic action of a system or components"*. This new category defines the functions of the real sensor that must be covered by the simulation. In order to systematically select functions, PerCollECT cannot be used in the same manner as for effects and causes, as this category is not yet included. To initially identify sensor functions, methods for the functional decomposition of sensors can be applied, for instance by building upon the decompositions proposed by [39,42]. Subsequently, it is possible to extend PerCollECT with the identified functions by systematically analyzing their relationships to existing effects and causes. Alternatively, one could first select effects and causes from PerCollECT and then determine which of the identified functions are required to model the effects and causes. In addition, the functions must be selected in such a way that the model delivers a validatable output. This requirement is explained in more detail in Section 2.3 and Section 3.1.1. As with the effects and causes, the functions to be incorporated in the simulation are also influenced by the external requirements. Furthermore, the selection of effects and causes also affects the selection of functions. In the context of a lidar simulation, for example, the functions distance or intensity measuring could be required. If the customer needs within the external requirements, that the output of the simulation only includes detections without intensity values, since these are not processed by the downstream SuT, the function for intensity measuring does not need to be implemented. However, if additional effects or causes are required that depend on measuring intensity, the function is nonetheless set as a requirement. In general, all the functions that are needed to implement the selected effects and causes are required.

In summary, the internal requirements can be defined as a specific collection of effects, causes, and functions that are to be implemented into the sensor simulation. After defining the external and internal requirements, the next step is to derive corresponding test cases.

#### 2.2.2. Derivation of Test Cases

**Figure 2:**
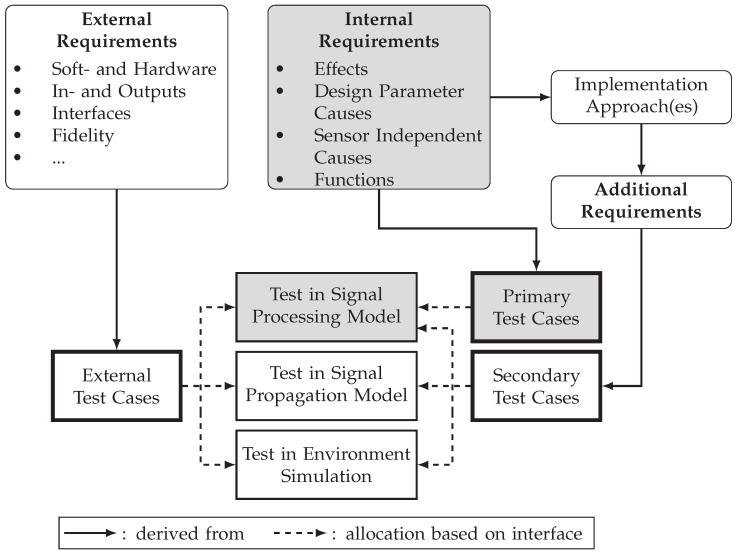
Method for derivation of test cases from the sensor simulation requirements.

Test cases are used to systematically assess if the previously defined requirements are met. Based on the definition provided in ISO/IEC/IEEE 24765:2017 [81] (p. 463), in general, a test case is a *“set of test inputs, execution conditions and expected results"*. In the context of this work, the test cases serve to ensure a structured, traceable and efficient V&V process by organizing the requirements into a checkable format. The subsequent section describes a method for systematically deriving test cases from internal and external requirements. Figure 2 outlines this method and indicates the simulation components in which the respective test cases are to be evaluated. Starting from the external and internal requirements for the sensor simulation, three different types of test cases can be defined: Primary, secondary and external test cases. Primary test cases are derived directly from internal requirements and are used to verify and validate them, for example, individual sensor effects, using quantitative metrics. Test cases are generally allocated to specific components of the simulation to enable their evaluation during development. The allocation of primary test cases is based on the corresponding interfaces of the effects, causes, and functions. Since all real sensor interfaces are located within the signal processing model in the applied simulation architecture, all selected effects, causes, and functions must be observable there. Consequently, all primary test cases are evaluated within the development of the signal processing model. At least one primary verification test and one primary validation test are derived for each effect, cause, or function. An exception applies to effects, causes, and functions that cannot be separated from each other, which will be addressed later in more detail. Secondary test cases, in contrast, are deduced from the additional internal requirements that result from the implementation approach(es) applied. An implementation approach in the context of this work refers to the technical solution used to realize one or more effects, causes and functions within the sensor simulation [77] (p. 31).

Additional requirements for the environment simulation and the signal propagation model arise, for example, from modeling the intensity measuring function of a lidar with a ray tracing-based implementation approach. For this purpose, the environment simulation must, for example, provide appropriate material parameters for the objects. The signal propagation model must be able to simulate the interaction between laser light and different materials. These additional requirements, which are checked by the secondary test cases, therefore depend on the selected implementation approach. This dependency does not exist for primary test cases, as these refer directly to the selected effects, causes, and functions, whereby it is not important how these ones are implemented. Since they are not directly derived from specific effects, causes and functions, secondary test cases may be tested in all three components of the simulation. Primary and secondary test cases are either specified for the verification or the validation of requirements. The external test cases are derived directly from the external requirements and are checked across all components of the sensor simulation. An example of this would be checking whether all three components are packaged as valid Functional Mock-up Units according to the Functional Mock-up Interface standard. In reference to the credibility levels defined by Ahmann et al. [70], it should be ensured that the combination of primary, secondary, and external test cases leads to each component of the sensor simulation fulfilling the criteria for all credibility levels.

This work focuses on sensor-specific properties within the simulation. Since a detailed analysis as well as a later development and V&V of all simulation components is not feasible, the following sections are primarily concerned with the signal processing model. Thereby, particular attention is being paid to the primary validation test cases. From the perspective of validating perception sensor simulations, the primary validation test cases are of particular interest, as they provide direct information about the validity of implemented sensor effects, causes, and functions, and thereby the sensor simulation. The demonstration of the proposed development method in Section 3 is based on the assumption that all external and secondary test cases are fulfilled and that the environment simulation and signal propagation model are valid. Figure 2 shows the elements in focus marked in gray. In the next section, the primary validation test cases are discussed in more detail.

#### 2.2.3. Taxonomy and Derivation of Acceptance Criteria for Primary Validation Test Cases

Given the emphasis on primary validation test cases, they will be discussed in more detail below. First, a new taxonomy for structuring the primary validation test cases is introduced. Subsequently, possibilities for deriving acceptance criteria for the individual test cases are discussed.

**Figure 3:**
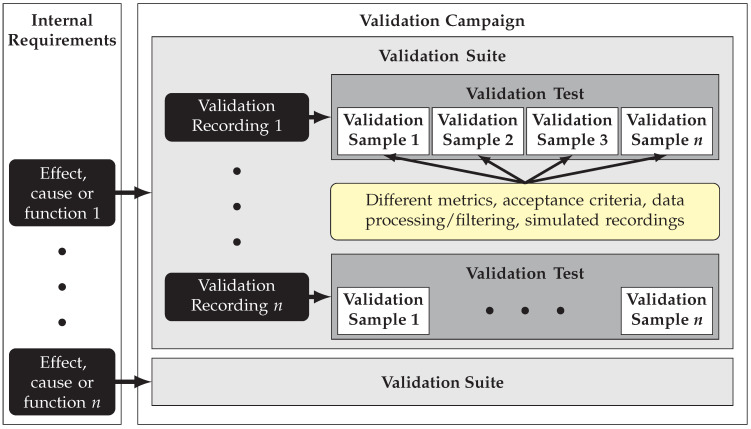
Illustration of taxonomy for structuring primary validation test cases.

The validation of specific effects, causes, and functions may involve numerous test cases, possibly based on different validation measurements and measurement setups. Therefore, a taxonomy is essential to ensure traceability and manage complexity throughout the development process. In addition, test cases must be organized in a structured manner to enable automated testing. The proposed taxonomy, visualized in Figure 3, is designed as follows: A validation campaign serves as the highest-level structure. It incorporates all primary validation test cases and their configuration which are defined within the validation process. A validation campaign includes multiple validation suites. Each validation suite targets a specific effect, cause or function related to the sensor. In Section 3.2.1, for example, a validation suite for the effect of distance noise is defined and analyzed. These encompass the description of the measurement setups, parameters, and other metadata that apply to the analysis of the specific effect, cause, or function. This ensures consistency and standardization throughout the validation process. Each validation suite consists of at least one validation test. A validation test corresponds to one specific validation recording of one sensor. The most granular element in the proposed structured taxonomy is a validation sample, which is equivalent to a single primary validation test case. In line with the definition of a test case, every validation sample must specifically include a validation and a simulated recording, along with a validation metric and an acceptance criterion. Furthermore, data processing steps such as filtering for individual objects or detections can also be part of a sample. A validation test can consist of multiple validation samples. Different validation samples within the tests differ in the application of different metrics and/or different acceptance criteria. Additional validation samples arise when different objects of a measurement setup are to be examined separately using different data processing steps. However, this option will not be relevant in the subsequent sections of this work, as the measurements always involve only a single object. If multiple simulated recordings are compared to a specific validation recording within a validation test, the resulting comparisons are assigned to separate validation samples as well. In general, the proposed taxonomy enables a systematic approach for the description of primary validation test cases.

A crucial part of the validation samples is the acceptance criterion according to which the samples are deemed to have passed or failed. In order to be able to justify these acceptance criteria in a reasonable way, an empirical approach for deriving quantitative acceptance criteria will be investigated in the further course. In this approach, a specific effect, cause, or function for which acceptance criteria are to be derived is first observed in the output data of multiple units of the same real sensor [77] (p. 31). Next, the differences in the effect, cause or function across the various sensor units are analyzed. It is expected that slight variations will be noticeable. For the simulated effect, cause, or function, it is therefore required that it falls within the range of these variations observed among the real sensors. In essence, this means that any deviations observed between identical real sensor units is also permissible for the sensor simulation. This empirical approach will be applied and further discussed in the demonstration of the proposed development method in Section 3. Another possible approach along the lines of Holder et al. [54], which is not further explored within the scope of this work, would be to apply a sensitivity analysis. Such an analysis would aim to assess the impact of deviations between the simulated and real effect, cause, or function on the SuT, allowing for the definition of acceptance criteria.

#### 2.2.4. Data Acquisition for Development and Validation of the Simulation

For the development and validation of sensor simulations, real sensor data is required. On the one hand, for calibrating the simulation and, on the other hand, as part of validation as a benchmark against which the output of the simulation is compared. Therefore, real-world measurements are conducted to collect data. In order to generate comparable simulated data, according to the fourth stage of the methodology outlined by Rosenberger et al. [9] (pp. 10–11) and Viehof [69] (p. 47), the real measurements must be monitored with additional reference sensors. The reference data, which includes, for example, the position and orientation of objects during the measurement, is then used to replicate the real measurement setup in a virtual environment for re-simulation. To ensure that the captured real and reference data as well as the simulated data provide a reliable basis for the development and validation process, it is necessary to validate the obtained data [9] (p. 10). This aspect of validating data is not described in detail by Rosenberger et al. and, to the best of the authors’ knowledge, no established overall approaches exist in the literature specifically in the context of the validation of perception sensor simulations. Therefore, the following section proposes an initial practical approach for validating real, reference, and simulated data.

**Figure 4:**
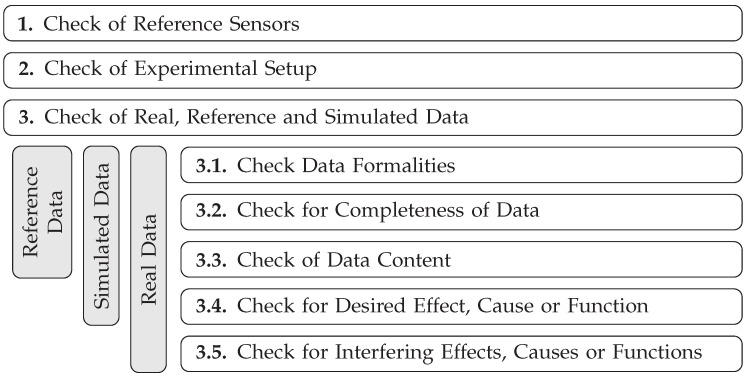
Proposed method for the validation of real, reference and simulated data.

Figure 4 illustrates this approach, which includes various sanity and plausibility checks of the collected data as well as the measurement setup and reference measurement equipment. In the context of this work, a sanity check examines whether something can be correct in principle. A plausibility check then additionally assesses whether the result also appears realistic. Prior to the real measurements, it is necessary to check the reference sensors that will be used. For this step, the method proposed by Holder et al. [82] can be applied to calibrate reference sensors, as exemplified for global navigation satellite system-based reference data in their work. To assess its trustworthiness, the laser range finder later used as a reference sensor in this work is checked in a separate test by comparing its measured distance values with those from another unit of the same type and a device from a different manufacturer. In the second step, the measurement setup is checked. The purpose is to confirm the consistency between the actual and the designed measurement setup, ensuring, for example, that the perception sensor itself as well as all other objects are precisely positioned and aligned. In this work, the task is carried out using the previously checked laser range finder in combination with an additional alignment laser. In the third and final step, which includes several sub-steps, the real, reference and simulated data is finally checked. It is important that the reference data is reviewed before the real measurement is re-simulated to generate the simulated data. To begin with, the data is checked to ensure that it is in a valid and correct file format.

The following step checks whether the data is complete, for example by evaluating the number and size of files or the number of captured frames. In the third sub-step, the content of the data is examined. This includes verifying whether a given recording file actually contains the intended validation data and whether the objects and their positions are correct. Comparing object positions between real and reference data serves as a plausibility check. These first three sub-steps are carried out for the real, reference, and simulated data. Next, the real and simulated data is examined to determine whether the effect, cause, or function to be validated is present. Finally, only real data is checked for any interfering effects, causes, or functions. With the acquisition of the real and reference data, the preparatory steps are concluded. The subsequent section introduces the proposed development method.

### 2.3. Development Method for Sensor Simulations

The development method for sensor simulations, introduced in this section, uses the results and data obtained during the preparatory steps as its input. The underlying idea of the method is to isolate the individual effects, causes, and functions specified in the requirements as clearly as possible and implement them iteratively one after the other into the simulation [77] (pp. 33–36). Each iterative change is followed by V&V of the simulation, enabling continuous assessment throughout the development process. This approach makes the entire process transparent and traceable, and after each development iteration a validated simulation is available that can already be used, for example, to support the development of other functions of an automated driving system. In addition, this approach offers a high degree of flexibility, since further effects, causes, and functions that are compatible with the implementation approaches already employed can be easily added at a later stage.

**Figure 5:**
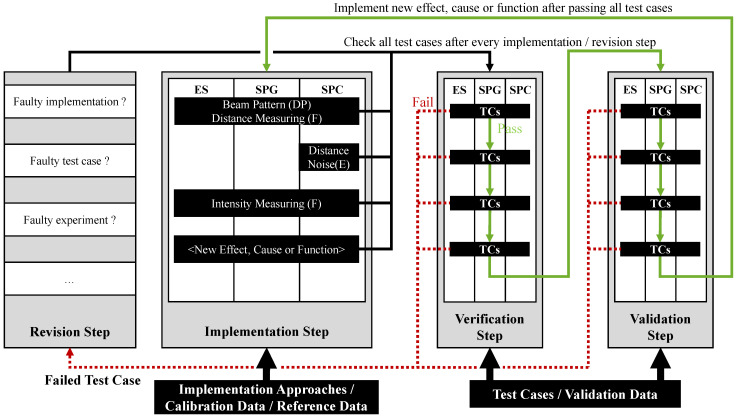
Iterative development method for sensor simulations with continuous V&V. The effects (E), design parameter causes (DPs), and functions (F) shown are to be considered examples. Test cases are abbreviated as TCs. Based on [77] (p. 34).

Based on this idea, the method, illustrated in Figure 5, consists of the following four distinct steps: Implementation step, verification step, validation step and revision step [77] (p. 33). Each iteration begins with an implementation step, in which ideally a single isolated effect, cause, or function is added to the simulation based on the corresponding implementation approach. If needed, it is calibrated using calibration data, and the simulation is then executed to generate the simulated data required for V&V. Effects, causes, or functions that cannot be separated from each other, meaning that they cannot be observed separately in the validation data, are jointly incorporated into the simulation in a single implementation step. Once the implementation step is complete, a verification step follows, during which all verification test cases derived for the effect, cause, or function are executed. After all test cases in the verification step are passed, a validation step follows in which all corresponding validation test cases are checked. Non-separable effects, causes, and functions are only tested by a single validation suite in the validation step. In each iteration, it must be assessed whether the implementation addresses a requirement linked to a secondary or external test case. When this is the case, the corresponding test cases must be checked within the same iteration. Provided that both the verification and validation steps are successfully completed without any failed test cases, the process continues with the next iteration where a new effect, cause, or function is implemented. As a result, the simulation is iteratively extended with additional effects, causes, and functions and continuously verified and validated. In each iteration, the test cases of all previous iterations are re-checked to ensure that new implementations do not interfere with existing ones. The sequence in which effects, causes, and functions are implemented must be reasonably defined by the developer, considering all requirements and the chosen implementation approach(es). Particular attention must be paid to the interdependencies. For effects, causes, and functions that depend on others, it must be ensured that the prerequisite effects, causes and functions are implemented in a previous or the same iteration. After the very first implementation step, it must be ensured that the simulation is able to generate a verifiable output which can be validated. In practice, this means that simulated output data can be used to check the test cases, for instance, by calculating certain metric values. Whenever a test case fails, a revision step is initiated to identify the reason for the failure and to eliminate it. Apart from a faulty implementation or calibration, possible reasons could also be faulty requirements or test cases. After the error has presumably been corrected in the revision step, the verification and validation steps are repeated before proceeding with the next iteration.

While Figure 5 shows the method using the simulation architecture for lidar sensors applied in this work, the method itself is also applicable to radar sensor. To apply the method to radar simulations, their specific effects, causes, and functions must be defined, followed by deriving corresponding test cases. Furthermore, other simulation architectures with differing interfaces and standards may need to be taken into account. The method is not tied to specific interfaces, and the architecture only determines, in combination with the implementation approaches, the component(s) of the simulation in which the test cases are implemented and assessed. In the given example, the components available are the environment simulation, the signal propagation model, and the signal processing model. As the number of test cases increases with each extension of the simulation and all previous test cases are re-tested in every iteration to avoid incompatibilities, automated testing becomes essential. To support this, the taxonomy defined in Section 2.2.3 can be used, as it helps to structure the test cases in a way that facilitates automation.

In summary, unlike the underlying methodology by Viehof [69] (p. 47) and Rosenberger et al. [9] (pp. 10–11), the iterative method presented here, together with the preparatory steps, explicitly encompasses the entire development process rather than focusing only on V&V. In other words, it constitutes a development method with integrated V&V rather than solely a method for V&V. Accordingly, the connections between requirement definition, the implementation of the simulation model, and the final V&V are examined in more detail. The approach therefore not only demonstrates how the model can be verified and validated, but also how it can be implemented in a systematic and traceable manner. Furthermore, concrete and practical methods and approaches for executing the entire development process are provided, with particular emphasis on opportunities for automation and scalability. Finally, the iterative approach enables a new level of flexibility, as additional findings and conditions, such as further measurement data, modified requirements, or new acceptance criteria, can be integrated iteratively at a later stage.

## 3. Exemplary Execution of the Development Method for a Lidar Sensor Simulation

The introduced method is demonstrated by exemplarily developing and validating a physical-based and high-fidelity lidar sensor simulation, specifically representing an Ouster OS1 32-layer lidar sensor. The simulation is validated on the point cloud interface, as this is the only accessible interface of the real sensor. As explained earlier, the focus of this work lies on the primary validation test cases; therefore, all other test cases are assumed to have been passed and the environment simulation as well as the signal propagation model are considered valid. Since the primary aim of this section is to demonstrate the method on a real-world example, further necessary simplifying assumptions are made, without which a demonstration would not be feasible. As a result, the developed simulation will have a rather small valid scope. However, extending this valid scope lies beyond the focus of this work and is identified as a subject for future research. In accordance with the considerations discussed in Section 2, the preparatory steps are addressed first. This is followed by the development and validation of the actual lidar simulation, carried out in two iteration steps.

### 3.1. Preparatory Steps for the Development of an Exemplary Lidar Sensor Simulation

In the following, the preparatory steps previously discussed are carried out exemplarily. However, due to the empirical approach used to determine acceptance criteria, data acquisition takes place prior to the derivation of test cases.

#### 3.1.1. Definition of Requirements for Lidar Sensor Simulation

In this section, some exemplary effects, causes, and functions are defined in the context of internal requirements, which are later realized in the simulation based on the proposed development method. First, effects, causes, and functions must be selected that guarantee an output which can be validated after the first iteration. Since the validation process involves comparing simulated point cloud detections with real sensor detections, the lidar simulation must be able to output detections in the form of point clouds after the first iteration. As a detection requires at least a distance as well as an azimuth and elevation angle, the function distance measuring and the design parameter cause beam pattern are selected first. The function of distance measuring is used to determine the distance value of the detection, and the beam pattern defines the corresponding azimuth and elevation angle. A preliminary analysis of real data revealed that both are inseparably connected with another effect. In the distance values of the real sensor, a distance offset is detected in comparison to the range finder used as reference sensor. Since the distance offset can be observed over different recordings and the reference sensor has been checked, a malfunction of the reference sensor is excluded and the offset is considered a sensor effect. Given that the function of distance measuring cannot be observed separately from the distance offset effect, they are considered inseparable and will be referred to hereafter as distance measuring and offset.

**Figure 6:**
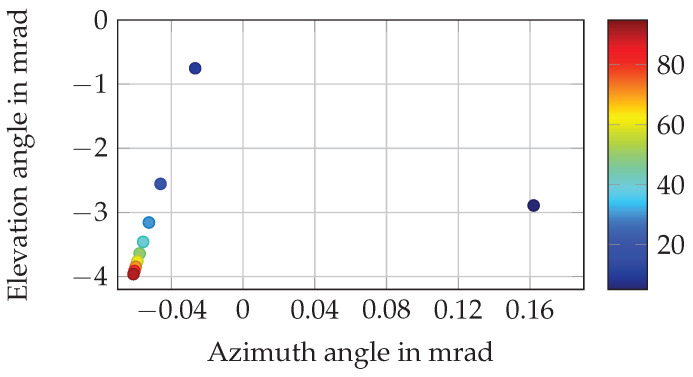
Deviation in azimuth and elevation angle of detections of a single beam. The distance of the detections in m is denoted in color.

On closer observation of the beam pattern, it is found that detections change slightly in azimuth and elevation angle depending on the distance value. This effect of distance dependent deviation of the beam pattern is illustrated in Figure 6. It arises from a positional offset between the lidar front optics, where the laser beam is physically emitted, and the lidar’s internal coordinate frame, in which the point cloud data are represented. As a consequence of the required transformation, the azimuth and elevation angles of individual detections exhibit slight shifts that vary with the measured distance. Further details can be found in the firmware user manual of the sensor [83] (pp. 20–25). Again, the design parameter cause beam pattern and the effect distance dependent deviation of the beam pattern cannot be observed separately, which is why both are considered inseparable and will be referred to jointly as beam pattern- and distance-dependent deviation in this paper. Since every measured value is subject to a random and a systematic error [84], the effect distance noise is further selected to approximate the real sensor behavior. The fourth and final effect, beam pattern noise, is chosen because it arises from the distance-dependent variation of azimuth and elevation angles in the beam pattern and is therefore directly induced by the distance noise effect. While the effect beam patter noise is dependent on the effect distance noise, both can be observed separately. In summary, the following four effects, causes and functions are defined for implementation:

- Distance measuring and offset.
- Beam pattern and distance-dependent deviation.
- Distance noise.
- Beam pattern noise.

At this point, it is noted that a full validation of the beam pattern, in particular due to the distance dependency, is not practically feasible within the scope of this work. To investigate all beams of the beam pattern under identical conditions, such as the distance to the target object and the angle of incidence on the object, would require highly sophisticated measurement setups, especially since the sensor has a field of view of 360° horizontally and 28.7° vertically. For this reason, based on the approach of Rosenberger [8] (pp. 123–132), a single beam will initially be considered within the scope of the validation. Since the distance-related effects and functions can also be examined and validated using just a single beam, this approach is suitable for all of the selected effects, causes, and functions. The following section clarifies which particular beam is used during validation and how the validation of the entire beam pattern can be practically achieved in the future by automating the real measurements. The only external requirement is that all implementations must be carried out within the presented simulation architecture.

#### 3.1.2. Data Acquisition

With the following measurements, validation data, necessary calibration data and data for deriving acceptance criteria as well as corresponding reference data are collected. The measurement setup is designed to ensure that all previously selected effects, causes, and functions are observable in the validation data, with the simplification that validation is initially limited to a single beam of the beam pattern. Furthermore, care is taken to minimize the influence of external factors such as ambient light or air humidity.

**Figure 7:**
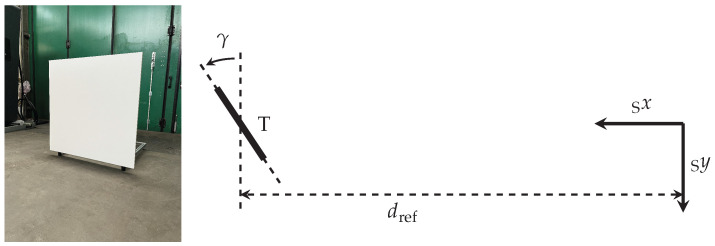
(**Left**): Square lidar target. (**Right**): Bird’s eye view of the measurement setup. The S-coordinate system denotes the origin of the lidar sensor and γ the yaw angle of the target T.

As a result, the measurement setup illustrated in Figure 7 is chosen. It consists of the lidar sensor mounted on a test rig and a square lidar target T with an edge length of 1 m. The target, which is positioned perpendicularly with a yaw angle of γ=0° to the x-axis of the sensor’s S-coordinate system at a reference distance $d_{\mathrm{ref}}$, features a near Lambertian diffuse reflectance factor of 80%. In the course of the measurements, the target is positioned at ten different distances, which are listed in Table 1. To minimize the influence of external factors, the measurements are carried out indoors at night in an approximately 95 m long corridor within a building of the Technical University of Darmstadt. Due to the length of the available corridor, the target distance is limited to a maximum of 90 m and the individual distances are selected to evenly cover the entire distance range. In order to obtain the necessary reference data for the re-simulation of the real measurements, the distance $d_{\mathrm{ref}}$ to the lidar target is determined using a Bosch GLM 120 C Professional laser rangefinder (Robert Bosch Power Tools GmbH, Stuttgart, Germany). According to the data sheet, the distance uncertainty of the laser rangefinder *u* is specified as ±(1.5 mm + 0.05 mm/m ·*d*), which will be considered in the re-simulation of the measurement setup [85], where distance *d* is calculated as $d=d_{\mathrm{ref}}-d_{\mathrm{offset}}$, since the origin of the range finder is offset by $d_{\mathrm{offset}}$ = 54.5 mm in front of the lidar along its x-axis. To ensure that the vertical axes of the sensor and the target are parallel to each other, both are leveled using a spirit level. Furthermore, to make sure that the rotation angle of the target γ is as close to 0° as possible, the target is centered along the x-axis of the sensor with the help of an alignment laser. Subsequently, the distances from the sensor’s origin to the two outer edges of the target are measured using the laser rangefinder. If these two distances are equivalent, the target is aligned perpendicular to the sensor’s x-axis. This procedure is based on the setup of Kirchengast and Watzenig [86] (p. 9376). Minimal deviations of the rotation angle due to the distance uncertainty of the rangefinder are neglected for simplicity due to the near Lambertian behavior of the target.

This target alignment, which is repeated for each reference distance, ensures that at least the beams close to the center of the beam pattern always lie on the target across all distances. For this reason, the center beam, whose detections are closest to 0° azimuth and 0° elevation angle, is therefore selected as the single beam for the validation of the selected effects, causes, and functions. Furthermore, this way, the angle of incidence on the target is kept as perpendicular as possible. In practice, the center beam detections are extracted for each frame from the point clouds using geometric filters.

**Figure 8:**
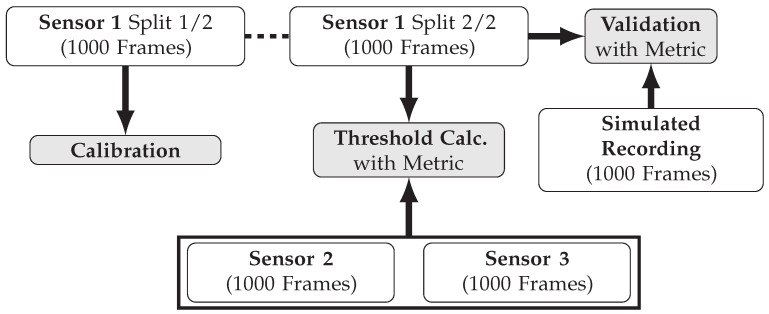
Utilization of the captured real lidar sensor data. The splitting is identical for each target distance and recording.

To efficiently obtain data for validation, calibration, and the empirical determination of acceptance criteria within the same real measurements, the following procedure is applied. At each target distance $d_{\mathrm{ref}}$, which is measured with the reference range finder, three identical Ouster OS1 sensors (further referred to as Sensor 1, 2 and 3) are employed one after another to capture a recording of 2000 point cloud frames each. The individual recording length of 2000 frames is selected to be as long as possible, constrained by the available time frame for the real measurements. The number of three sensor units results from the maximum number of units available to the authors. The sensors are positioned on the test rig using dowel pins, ensuring that the measurement setup remains identical for each sensor. Prior to utilization, all obtained data is reviewed according to the steps discussed in Section 2.2.4. The captured real lidar data serves three different purposes: Calibrating the sensor simulation, validating the sensor simulation, and determining the acceptance criteria. Figure 8 illustrates how these three purposes are fulfilled using the data.

The first split containing 1000 frames from each recording of Sensor 1 is used to calibrate the sensor simulation. Accordingly, the developed simulation features the characteristics of Sensor 1. The second recording split of Sensor 1 is used as validation data and is compared with the simulated data as part of the validation. As with the validation data split, a simulated recording consists of 1000 frames each. For Sensor 2 and Sensor 3, only the second half of the recorded 2000 frames are used for calculating the acceptance thresholds, ensuring that all recording splits compared are of equal length. A detailed breakdown of how the calculation of the acceptance criteria in the form of threshold values works is provided in the next section. The question of the ideal duration for validation and calibration recordings remains an open research topic.

One possible way to validate the entire beam pattern could be to automate the described measurements. To achieve this, the sensor would need to be rotated automatically, for example using a robotic arm, so that one of the beams always hits the target at a perpendicular angle. This would allow all beams in the beam pattern to be examined step by step under consistent conditions. Ideally, the positioning of the lidar target should also be automated to further accelerate the process. For future measurements, it is also advisable to monitor the temperature of the sensor directly to ensure a consistent performance.

#### 3.1.3. Exemplary Derivation of Validation Test Cases with Acceptance Criteria

To validate the previously defined requirements, validation samples for the selected effects, causes, and functions are derived in the following based on the taxonomy introduced in Section 2.2.3. Once these validation samples have been defined, the corresponding validation metric is explained and the process of calculating the acceptance thresholds is described.

##### Derivation of Validation Samples

According to the taxonomy, each of the four selected effects, causes, and functions requires an individual validation suite. Since each of them is to be validated with the same validation data, ten individual validation recordings at ten different target distances are available for each suite. This results in ten validation tests per validation suite. The number of validation samples per validation test is determined by the number of simulated recordings that are being compared to one validation recording and the number of parameters describing the effect, cause, or function. As part of this work, each of the ten validation recordings is re-simulated three times. This approach ensures that the uncertainty *u* of the laser rangefinder, which measures $d_{\mathrm{ref}}$, is accounted for in the validation process [8] (p. 126). Consequently, in the first simulated recording (Sim. 1), the target is positioned at the distance $d_{\mathrm{ref}}$ from the sensor. In the second and third simulated recordings (Sim. 2 and Sim. 3), the target is then placed once at a distance of $d_{\mathrm{ref}}$ plus the uncertainty *u* and once at a distance of $d_{\mathrm{ref}}$ minus the uncertainty *u*. These three simulations completely cover the uncertainty range of the reference sensor. The question of whether it would be useful to perform further simulations in the uncertainty range of the reference sensors is discussed later on.

Considering a single beam, the design parameter cause/effect beam pattern- and distance-dependent deviation and the effect beam pattern noise are each described by the two parameters azimuth angle α and elevation angle ε. For each of the two, the combination of three simulated recordings and two parameters yields six validation samples per validation test and, therefore, 60 validation samples per suite. In contrast, the function/effect distance measuring and offset and the effect distance noise are each characterized by the single parameter distance *d*. As a result, this yields three validation samples per validation test and 30 validation samples per suite.

##### Validation Metric

**Figure 9:**
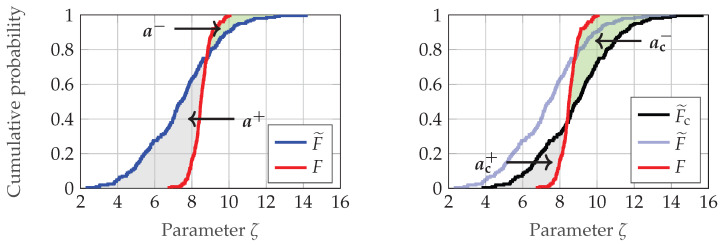
(**Left**): Calculation of model bias $m_{\mathrm{bias}}$. (**Right**): Calculation of model scattering error $m_{\mathrm{CAVM}}$. Based on [8] (p. 118) and [77] (p. 17).

For each validation sample, a metric must be defined to quantify the differences between the validation and simulated data with respect to a specific parameter, such as the distance. On the basis of the differences quantified using the metric, the acceptance criterion can then be used to decide whether the validation sample has passed or failed. In this work, the double validation metric (DVM) introduced by Rosenberger [8] (pp. 118–119) is used for this purpose, as it is applicable to all parameters of the defined effects, causes, and functions and offers an intuitive interpretation. The DVM quantifies the differences between two empirical cumulative distribution functions (EDFs). This involves determining the model bias, representing the mean deviation between the two EDFs, and the so-called model scattering error, which reflects the variations in the shape of the two curves [87] (p. 6). The model bias, introduced by Voyles and Roy [88] on the basis of the area validation metric of Ferson et al. [89], is calculated from the difference between the two areas $a^{+}$ and $a^{-}$ that are enclosed by two EDFs (see Figure 9).

The bias $m_{\mathrm{bias}}$ of the EDFs F and $\tilde{F}$ is therefore given by

(1) $$m_{\mathrm{bias}}\left(F,\tilde{F}\right)=a^{-}-a^{+}.$$

To calculate the model scattering error, the corrected area validation metric (CAVM) introduced by Rosenberger [8] (pp. 118–119) is used. Accordingly, one of the EDFs, in this case $\tilde{F}$, is shifted by the previously calculated bias $m_{\mathrm{bias}}$, resulting in the corrected EDF ${\tilde{F}}_{c}$. Next, similar to the calculation of the bias, the two areas $a_{c}^{-}$ and $a_{c}^{+}$ that are enclosed by the two EDFs F and ${\tilde{F}}_{c}$ are determined (see Figure 9). Finally, according to [8] (p. 118), the model scattering error $m_{\mathrm{CAVM}}$ is given by

(2) $$m_{\mathrm{CAVM}}\left(F,{\tilde{F}}_{c}\right)=a_{c}^{-}+a_{c}^{+}.$$

To generate the EDFs from the validation and simulated recordings, the detections of the center beam, which are extracted using geometric filters, are collected and aggregated over all 1000 frames. Depending on the effect, cause, or function, the EDF is then calculated for one of the corresponding parameters of these detections.

##### Empirical Derivation of Acceptance Thresholds

**Figure 10:**
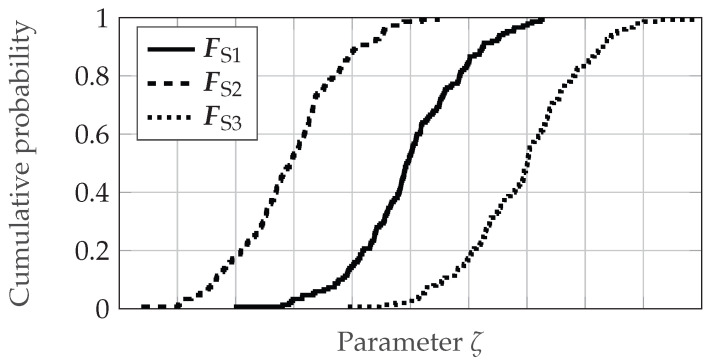
Exemplary illustration of the EDFs $F_{S1}$, $F_{S2}$ and $F_{S3}$ of the three sensors for a parameter ζ.

The presented metric is also used to empirically determine the acceptance thresholds for individual validation samples. As already described, the basic idea behind this empirical approach is that the deviations that can be observed in the data from several real sensors are also allowed for the simulation. This means that the simulation deviates from the real sensor to the same extent as the real sensor deviates from other identical real sensors. For this purpose, the data split also used for validation of Sensor 1 is compared with a split of the recordings of Sensor 2 and Sensor 3, as described in Figure 8. This comparison is performed individually for all ten target distances. Thus, for each distance, the differences across the three sensors are calculated for the three parameters distance, azimuth and elevation angle using the two metrics $m_{\mathrm{bias}}$ and $m_{\mathrm{CAVM}}$. For the recordings with a target distance $d_{\mathrm{ref}}$ and an unspecified parameter ζ (e.g., distance or azimuth angle), the EDFs $F_{S1}$, $F_{S2}$ and $F_{S3}$ of the three sensors could look like the ones depicted in Figure 10. The acceptance thresholds are derived from the comparisons of the EDFs $F_{S1}$ and $F_{S2}$ as well as $F_{S1}$ and $F_{S3}$.

Consequently, for validation samples in which a parameter ζ is examined using the metric $m_{\mathrm{bias}}$, the lower threshold $T_{\mathrm{low},m_{\mathrm{bias}}}$ is calculated as follows:

(3) $$T_{\mathrm{low},m_{\mathrm{bias}}}=\left\{\begin{array}{cc} 0.0 & \mathrm{for}\;m_{\mathrm{bias}}\left(F_{S1},F_{S2}|F_{S3}\right)>0,\\ \min(m_{\mathrm{bias}}\left(F_{S1},F_{S2}|F_{S3}\right)) & \mathrm{otherwise}. \end{array}\right.$$

The upper acceptance threshold $T_{\mathrm{up},m_{\mathrm{bias}}}$ yields

(4) $$T_{\mathrm{up},m_{\mathrm{bias}}}=\left\{\begin{array}{cc} 0.0 & \mathrm{for}\;m_{\mathrm{bias}}\left(F_{S1},F_{S2}|F_{S3}\right)<0,\\ \max(m_{\mathrm{bias}}\left(F_{S1},F_{S2}|F_{S3}\right)) & \mathrm{otherwise}. \end{array}\right.$$

As Equation (2) indicates, the threshold for the metric $m_{\mathrm{CAVM}}$ must be greater than or equal to 0, meaning that only an upper threshold needs to be defined. Consequently, the upper threshold $T_{\mathrm{up},m_{\mathrm{CAVM}}}$ is determined by

(5) $$T_{\mathrm{up},m_{\mathrm{CAVM}}}=\max\left(m_{\mathrm{CAVM}}\left(F_{S1},F_{S2}|F_{S3}\right)\right).$$

On this basis, acceptance thresholds are calculated for all validation samples, which will be presented in the next section along with the validation results. The question of whether the number of three sensor units is really sufficient to be able to set meaningful thresholds will be discussed in due course. Table 2 summarizes the parameters to be tested for the four selected effects, causes, and functions along with their corresponding metrics.

As the design parameter cause/effect beam pattern and distance dependent deviation and the function/effect distance measuring and offset represent angle and distance offsets, the metric $m_{\mathrm{bias}}$ is suitable for quantifying them. Given that differences in noise behavior are reflected in the shape of the EDFs and thus the scattering error, the metric $m_{\mathrm{CAVM}}$ is applied for effects distance noise and beam pattern noise.

In summary, an exemplary validation sample for the effect of distance noise is composed as follows. In this case, the parameter to be checked is the distance *d* and the metric to be applied is $m_{\mathrm{CAVM}}$. Furthermore, a concrete acceptance value $T_{\mathrm{up}}$ is calculated according to the approach described above. Depending on the validation test to which the sample belongs, a specific validation recording and one of the associated simulated recordings from the re-simulation are assigned to the sample. As a result, all necessary information is available in the subsequent validation step to compute the metric and determine whether the sample meets or fails the threshold.

### 3.2. Realization and Validation of Lidar Sensor Simulation

With the requirements and corresponding validation samples derived and the necessary validation, calibration, and reference data acquired, the following section presents the realization and validation of the exemplary lidar sensor simulation. Using the development method introduced in Section 2.3, two iterations are carried out. In order to be able to evaluate the large number of validation samples automatically and efficiently in the validation step, the validation software Avelon (Version 1.0.0) by Persival GmbH (Ober-Ramstadt, Germany) [90] is used. In Avelon, individual validation samples are configured using a graphical user interface. Predefined data loaders for the recordings, metrics, and acceptance criteria can be dragged to a canvas and connected to form the individual validation samples. In addition, a geometric filter can be set, which is used to extract the detections of the center beam from the point cloud.

#### 3.2.1. First Iteration

In the implementation step of the very first iteration, it is necessary to ensure that a validatable output is available on the basis of which the validation samples can be evaluated. Therefore, the design parameter cause/effect beam pattern- and distance-dependent deviation and the function/effect distance measuring and offset are jointly realized in the simulation in the first implementation step to enable outputting point cloud detections. To that end, a ray tracing-based implementation approach is used, in line with the simulation architecture introduced in Section 2.1. The ray tracing, which is the basis for the function/effect distance measuring and offset, is realized with the help of the SMDL provided by Persival GmbH. Based on the beam intrinsics of the real sensor [83] (pp. 86–87), the beam pattern including the center beam is initially implemented by parameterizing the ray tracer. Each laser beam emitted by the real sensor is traced by a single ray from the ray tracer. To account for the additional distance-dependent deviation connected with the beam pattern, the origin of the individual rays in the ray tracer is defined according to the actual sensor geometry, incorporating an offset relative to the internal coordinate system. The positional offset is also derived from the beam intrinsics of the real sensor [83] (pp. 86–87). Accordingly, the necessary coordinate transformation is added. It remains uncertain whether the laser beams of the real sensor are truly emitted as specified in the beam intrinsics. To determine this possible deviation and account for it in the simulation, another more complex measurement setup would be needed to accurately measure the actual emitted beam pattern. In the context of this work, a possible deviation of the actual beam direction from the specified one is considered as a further sensor effect that would have to be taken into account in later iteration steps of the development of the lidar simulation. The offset in the distance measuring is derived from the calibration recording splits of Sensor 1 (see Figure 8) as well as the reference data. For this purpose, the distance values of the center detection points are analyzed over all target distances and compared with the corresponding reference distance. Next, a mathematical function is fitted across all offsets as a function of distance to obtain a description of the behavior. The resulting function is used to realize the offset in the simulation. After the implementation and calibration is complete, the re-simulation of the measurements described in Section 3.1.2 must follow in the preparation of the verification and validation step. With the ten different target distances and taking into account the uncertainty of the laser range finder (see Section 3.1.3), a total of 30 simulated recordings, each with 1000 frames, are generated.

**Figure 11:**
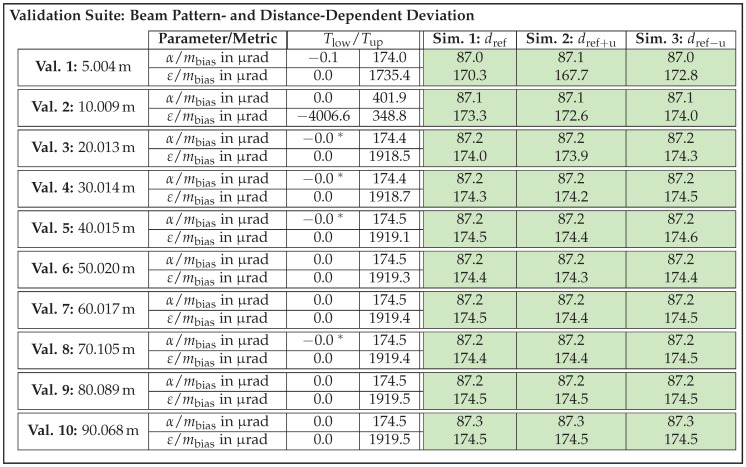
Validation result of the design parameter cause/effect beam pattern- and distance-dependent deviation in the first iteration. ^*^ While the threshold value appears as 0.0 when rounded to one decimal place, the actual unrounded value is slightly less than zero.

**Figure 12:**
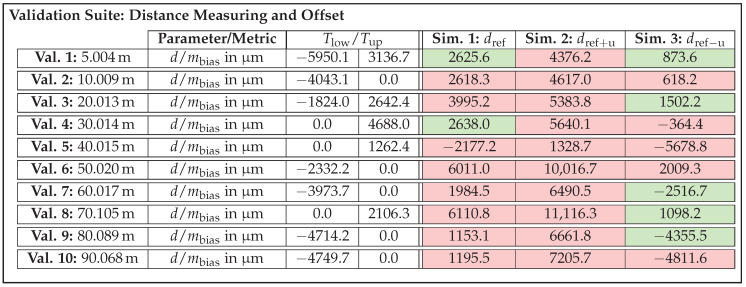
Validation result of the function/effect distance measuring and offset in the first iteration.

Assuming that the verification step has been successfully completed, the first validation step follows. In the first iteration, a validation suite for design parameter cause/effect beam pattern- and distance-dependent deviation and one for function/effect distance measuring and offset must be evaluated. The results of these validation suites are summarized in Figure 11 and Figure 12. Each of the green or red colored cells represents the result of a single validation sample, with green indicating a passed sample and red indicating a failed one. A validation sample is considered to have passed if the metric value calculated from the validation recording and simulated recording does not exceed the respective upper or lower thresholds. All validation samples calculated on the basis of a specific validation recording (e.g., Val. 1) constitute a validation test. The validation suite for design parameter cause/effect beam pattern- and distance-dependent deviation indicates that its implementation is valid under the given assumptions, as it comprises exclusively passed validation samples.

Conversely, the validation suite of function/effect distance measuring and offset shows the majority of failed validation samples (see Figure 12), which is why a revision step is initiated for further investigation. A comparison of the validation tests for Val. 1 and Val. 2 reveals that the $m_{\mathrm{bias}}$ values of the individual validation samples are quite similar. The $m_{\mathrm{bias}}$ values of Val. 2 exhibit a maximum relative deviation of approximately 29% and a maximum absolute deviation of 0.26 mm compared to those of Val. 1. However, the acceptance interval spanned by the thresholds of the validation samples for Val. 2 is 56% smaller in relative terms and 5.04 mm in absolute terms than that of Val. 1. Upon examination of all thresholds from Figure 11 and Figure 12, it is noticeable that those of function/effect distance measuring and offset differ significantly more across all recordings than those of design parameter cause/effect beam pattern- and distance-dependent deviation. For this reason, it is assumed that the failed validation does not result from the developed simulation itself, but rather from the empirically defined acceptance criteria. As the thresholds are derived solely from real sensor recordings, it is evident that the function/effect distance measuring and offset is significantly more random in nature. It is concluded that, in this case, the number of two comparative lidar sensor units used to calculate the thresholds is insufficient to derive meaningful acceptance criteria. As obtaining a statistically significant number of real sensor units for iterating the acceptance criteria is not feasible in the context of this work, an alternative approach is used to define the acceptance criteria in this case. Specifically, the distance accuracy of the real sensor as specified by the manufacturer is used and set as the acceptance criterion. Consequently, the simulated sensor is required to remain within this defined accuracy range. According to the data sheet of the real sensor [91], this results in the acceptance criteria of $T_{\mathrm{low}}=-3\;\mathrm{cm}\;\left(-30,000\;\mu m\right)$ and $T_{\mathrm{up}}=3\;\mathrm{cm}\;\left(30,000\;\mu m\right)$ for all validation samples. With the revision of the acceptance criteria, the revision step is considered complete and the validation step is repeated to assess the changes. Since no changes have been made to the simulation or the data, it is sufficient to review the validation suite of function/effect distance measuring and offset. Figure 12 shows that none of the $m_{\mathrm{bias}}$ values calculated for the individual samples exceed the redefined acceptance thresholds. The implementation is therefore considered valid under the assumptions made, allowing the continuation with the second iteration.

#### 3.2.2. Second Iteration

The second iteration starts with another implementation step. This time, the two effects distance noise and beam pattern noise are incorporated simultaneously into the simulation. Ideally, a separate iteration would be executed for each effect. However, due to the following factor, they are both implemented within a single iteration. It is expected that the distance dependence of the beam pattern, implemented in the previous iteration, will lead to corresponding beam pattern noise once the distance noise is incorporated in the simulation. This means that by implementing the distance noise, the beam pattern noise is also inevitably implemented. Consequently, no further implementation step or iteration is necessary.

To realize distance noise in the simulation, a normally distributed noise is applied to the distance values calculated by the ray tracer. Whether the noise of the real sensor is in fact normally distributed will be assessed during the validation step. The distance noise is also modeled as a mathematical function of the distance to the target and is thereby distance-dependent. To obtain this mathematical description, the calibration recording splits of Sensor 1 are used again and the detection points of the center beam are evaluated for each target distance. Next, the standard deviation of the distance values is calculated for each target distance and, subsequently, a corresponding function is fitted that describes the correlation between the standard deviation and the target distance. According to the fitted function, the standard deviation of the distance noise increases with a larger target distance. Identical to the previous iteration, the extended lidar simulation is employed in preparation of the following steps to re-simulate the measurements and obtain corresponding simulated data.

**Figure 13:**
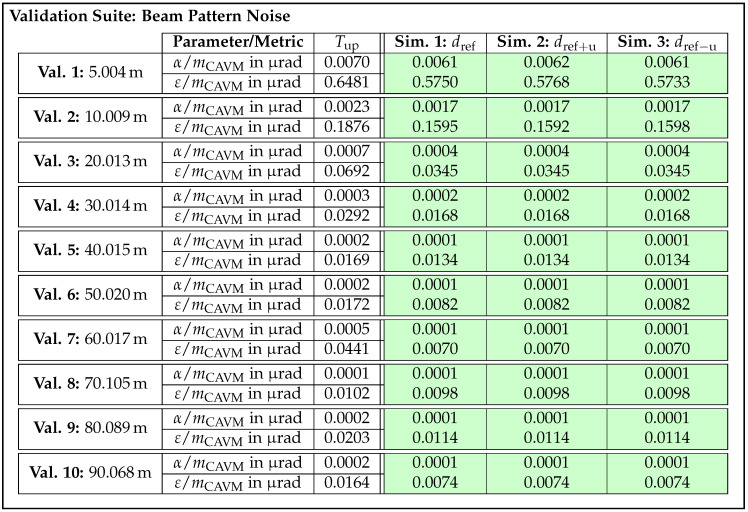
Validation result of the effect beam pattern noise in the second iteration.

**Figure 14:**
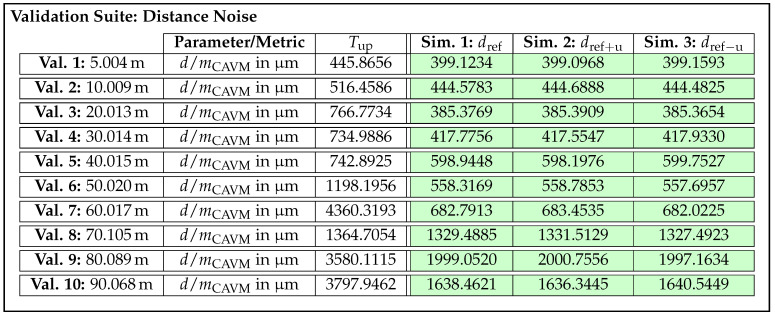
Validation result of the effect distance noise in the second iteration.

Assuming once again that the verification step has been passed, the validation step follows. Within the validation step of the second iteration, a total of four validation suites are evaluated, one for each effect, cause, and function implemented so far. The two validation suites from the first iteration are re-calculated using the simulated data of the current iteration to ensure that the newly implemented effects do not compromise the validity of the previous implementations. Since re-evaluating the validation suites shows no differences in passed or failed validation samples compared to the previous iteration, the results are not presented in detail. The validation results of the newly implemented effects distance noise and beam pattern noise, however, are summarized in Figure 13 and Figure 14. It can be concluded that in both validation suites, all validation samples have successfully passed. As the metric $m_{\mathrm{CAVM}}$ is used instead of $m_{\mathrm{bias}}$ in both validation suites, only an upper threshold is defined. The successful validation result suggests that the assumptions made during the implementation step are justified. In particular, the assumption that distance noise follows a normal distribution has, until now, been considered uncertain. With the successful completion of the validation step, the second iteration is concluded, allowing the simulation development to proceed with the implementation of the next effect, cause or function. However, in the context of this work, the development process is discontinued at this point. Future steps and open questions will be addressed in the following discussion.

## 4. Discussion

As demonstrated in Section 3, the method can be used to develop sensor simulations in a credible and traceable way. The following section provides a discussion of the proposed development method and its corresponding preparatory steps as well as the exemplary application.

The proposed development method offers several advantages. By iteratively implementing isolated effects, causes, and functions that are directly derived from the internal requirements, the overall simulation development process becomes highly structured and traceable. Due to the continuous V&V approach, it is guaranteed that a valid sensor simulation is available after each iteration. Moreover, this facilitates troubleshooting and enables subsequent extensions of the simulation without compromising its validity. However, a drawback related to the iterative approach is the increasing number of test cases that must be assessed with each iteration, as all test cases from previous iterations need to be re-evaluated. Therefore, automating the evaluation of test cases is essential for maintaining development efficiency. In general, it would be desirable to automate as many aspects of the development process as possible. In practice, this could be achieved using continuous integration and continuous delivery pipelines, for example. Although this involves higher initial expense, these are offset during the development process.

Furthermore, it must be emphasized that the lidar sensor simulation developed in this work is intended primarily as a proof-of-concept to illustrate the proposed development process and methods. In order to use the simulation for validating complex ADAS and AD functions, its valid scope must be significantly extended and it must be shown that it generalizes to different setups. This requires, on the one hand, the incorporation of additional effects, causes, and functions and, on the other hand, the execution of further validation measurements. For instance, the subsequent step may be the implementation of the intensity measuring function, thereby providing the foundation for modeling effects such as false-negative detections. Regarding the validation measurements, the next step could be to employ additional targets with varying materials and reflectivities, as well as to vary the orientation of both the sensor and the target. This allows, on the one hand, an extension of the valid scope to the entire beam pattern and, on the other hand, the validation of the intensity-measuring function for different materials, reflectivities, and incidence angles. However, this also requires a high degree of automation of the measurements, which therefore proves to be essential for an efficient validation process. Initially, the measurement setups defined in the DIN SAE SPEC 91471:2023-05 [74], which describes an assessment methodology for automotive lidar sensors, might be used. In the standard, in addition to a target with a reflectance factor of 80%, targets with reflectance levels of 50% and 10% as well as a retro-reflector are utilized [74] (p. 24). In the context of ADAS and AD, certain sensor effects, causes, or functions may only become apparent in more complex environments or traffic situations. Therefore, more complex validation measurements and setups are required for extending the sensor simulation. For example, to include the motion scan effect [92] into the valid scope, it is necessary to move from a purely static to a dynamic measurement setup. To account for weather effects and environmental influences, it is necessary to transition from highly controlled indoor setups to configurations in which the sensor is exposed to these conditions. In addition to the purpose of expanding the valid scope, it is also beneficial to carry out additional measurements with appropriate automation in order to facilitate the detection of outliers and measurement errors, for example, as part of data validation. Furthermore, it is important to justify the selection of the additional effects, causes and functions in a reasonable way. The CEPRA method by Linnhoff et al. [56] (p. 5) mentioned earlier can be used for this, which decides on the basis of two experts which effects and causes are particularly relevant for a given SuT. However, downstream SuTs such as modern automated driving functions are becoming increasingly reliant on deep learning and artificial intelligence [93], which are often difficult for humans to interpret due to their black box character [94]. In such cases, a data-driven approach using sensitivity analysis may offer a more appropriate solution and should therefore be investigated in the future. Additional simplifications that are not permissible in a real-world application of the method include the assumptions that all verification test cases have been passed and that the environment simulation and the signal propagation model are valid.

Although it was shown that empirically deriving acceptance criteria is partially feasible for certain effects, no final conclusion can be drawn on this method due to the limited number of sensor units available. As discussed in Section 3.2.1, three sensors are not enough to ensure the statistical robustness needed to establish meaningful thresholds for one of the validation suites. In principle, the method can be applied to all effects, causes and functions whose descriptive parameters vary from sensor unit to sensor unit under identical measurement setups, thereby yielding a probability distribution. Ideally, the parameter values should exhibit a low variance across different sensor units so that, as in the determination of confidence intervals [95] (p. 176), a stable distribution emerges even for small sample sizes (in this case, the required number of units). In the context of calculating confidence intervals, in the literature, a sample size greater than 40 is suggested [95] (p. 177), although it is also noted that an exact determination is difficult due to the unknown variance [95] (p. 182). From a developer’s perspective, minimizing the required number of sensor units and measurements is desirable in order to reduce the overall effort. With the objective of demonstrating that the simulation lies within the deviation range of real sensors, it may be reasonable to begin with a smaller number of units and to include additional units only in cases where failed test cases are suspected to be caused by non-meaningful thresholds. In conclusion, further research is necessary to enable more concrete statements regarding the applicability of the method and the necessary number of sensor units. Although this approach may be less restrictive for sensor manufacturers, as they have easier access to a larger number of sensors, its reliance on many units to determine meaningful thresholds remains a significant limitation. Furthermore, while this empirical approach enables deriving comprehensible acceptance criteria, the resulting thresholds appear to be rather conservative and strict. The fundamental question arises as to whether such a high level of simulation quality is actually needed or whether less restrictive acceptance criteria would be sufficient. It would therefore be reasonable to consider the SuT while determining the acceptance criteria in future work.

Another unresolved topic is how to deal with the uncertainties of the reference sensors that provide crucial information for the re-simulation of the measurement setup. In order to take the uncertainties of the reference sensor into account during the validation, several simulations are carried out. Future studies could extend the current approach by conducting additional simulations within the uncertainty range of the reference sensor. This could enable a more precise evaluation of the influence of reference sensor uncertainty on the validation results. In this context, a related problem is the increasing number of uncertain reference parameters in more complex validation measurements. In future measurements, it is conceivable that, in addition to the distance to the target, its position along the sensor’s y- and z-axes and orientation will become relevant. This leads to a fundamental challenge: as the number of uncertain reference parameters increases, the total number of required simulations increases significantly. Addressing this issue remains an open question for future research.

## Figures and Tables

**Table 1:** Identification numbers (IDs) of the measurements with corresponding distance to the target $d_{\mathrm{ref}}$. The uncertainty is calculated as u=±(1.5 mm + 0.05 mm/m · ($d_{\mathrm{ref}}-d_{\mathrm{offset}}))$.

ID	1	2	3	4	5	6	7	8	9	10
$d_{\mathrm{ref}}$ in m	5.004	10.009	20.013	30.014	40.015	50.020	60.017	70.105	80.089	90.068
*u* in mm	±1.7	±2.0	±2.5	±3.0	±3.5	±4.0	±4.5	±5.0	±5.5	±6.0

**Table 2:** Summary of the parameters required for validating the selected effects, causes, and functions and applied metrics. The three parameters are azimuth angle α, elevation angle ε, and distance *d*.

Effect/Cause/Function	Parameter	Metric
Beam pattern- and distance-dependent deviation	α	$m_{\mathrm{bias}}$
	ε	$m_{\mathrm{bias}}$
Distance measuring and offset	*d*	$m_{\mathrm{bias}}$
Distance noise	*d*	$m_{\mathrm{CAVM}}$
Beam pattern noise	α	$m_{\mathrm{CAVM}}$
	ε	$m_{\mathrm{CAVM}}$

## Data Availability

The real and simulated lidar data of this publication is available at https://tudatalib.ulb.tu-darmstadt.de/handle/tudatalib/4586, accessed on 6 June 2025.

## Abbreviations

The following abbreviations are used in this manuscript:

AD	Automated Driving
ADAS	Advanced Driver Assistance System
CAVM	Corrected Area Validation Metric
CEPRA	Cause, Effect, and Phenomenon Relevance Analysis
DVM	Double Validation Metric
EDF	Empirical Cumulative Distribution Function
ES	Environment Simulation
FMI	Functional Mock-up Interface
ID	Identification Number
ODD	Operational Design Domain
OSI	Open Simulation Interface
SMDL	Sensor Model Development Library
SPC	Signal Processing Model
SPG	Signal Propagation Model
SuT	System under Test
UNECE	United Nations Economic Commission for Europe
V&V	Verification and Validation
